# A Customized Neural Transcranial Magnetic Stimulation Target for Functional Disability Among Veterans With Co-Occurring Alcohol Use Disorder and Mild Traumatic Brain Injury: Protocol for a Pilot Randomized Controlled Trial

**DOI:** 10.2196/64909

**Published:** 2025-06-23

**Authors:** Amy A Herrold, Alexandra L Aaronson, Dulal Bhaumik, Timothy Durazzo, Sherri L Livengood, Alma Ramic, Patrick Riordan, Neil Jordan, Todd Parrish, Trudy Mallinson, Ibuola O Kale, Andrea Billups, Kelly Krese, Sandra Kletzel, Noah S Philip, Theresa L Bender Pape

**Affiliations:** 1 Edward Hines Jr, VA Hospital Hines, IL United States; 2 Department of Psychiatry and Behavioral Sciences Feinberg School of Medicine Northwestern University Chicago, IL United States; 3 Department of Psychiatry University of Illinois Chicago Chicago, IL United States; 4 Department of Epidemiology and Biostatistics University of Illinois Chicago Chicago, IL United States; 5 Sierra-Pacific Mental Illness Research and Education Clinical Centers VA Palo Alto Health Care System Palo Alto, CA United States; 6 Department of Psychiatry and Behavioral Sciences Stanford Medicine Stanford, CA United States; 7 Department of Physical Medicine and Rehabilitation Feinberg School of Medicine Northwestern University Chicago, IL United States; 8 Department of Radiology Northwestern University Chicago, IL United States; 9 Department of Clinical Research and Leadership George Washington University Washington, DC United States; 10 Shirley Ryan AbilityLab Chicago, IL United States; 11 Center for Neurorestoration and Neurotechnology VA Providence Health Care System Providence, RI United States; 12 Department of Psychiatry and Human Behavior Alpert Medical School Brown University Providence, RI United States

**Keywords:** transcranial magnetic stimulation, magnetic resonance imaging, alcohol, traumatic brain injury

## Abstract

**Background:**

Alcohol use disorder (AUD) and mild traumatic brain injury (mTBI) commonly co-occur, exacerbating symptoms and negatively impacting function. Co-occurring AUD and mTBI (AUD+mTBI) represents a unique and heterogeneous brain state impacting symptoms and function. Repetitive transcranial magnetic stimulation (rTMS) is a noninvasive neuromodulatory treatment with burgeoning evidence for improving brain function and symptoms for AUD and mTBI each alone. However, there is no consensus on the optimal neural target or treatment site of stimulation for either condition alone or when they co-occur.

**Objective:**

This study aims to (1) test the preliminary efficacy of high-frequency rTMS provided to a customized neural target to treat functional disability among veterans with AUD+mTBI and (2) assess the sustainability of rTMS effects on functional disability among veterans with AUD+mTBI.

**Methods:**

This single-blind randomized controlled trial (RCT) involved treatment-seeking veterans with AUD+mTBI recruited from a Department of Veterans Affairs hospital. Veterans will be randomly assigned to (1) a novel TMS target site using neuronavigation or (2) standard clinical left dorsolateral prefrontal cortex using the Beam F3 method. All participants first receive 10 daily sessions of sham rTMS, followed by 10 daily sessions of active rTMS (10 Hz) provided by a trained TMS administrator. Veterans will complete self-report study questionnaires before and after sham and active rTMS session blocks as well as at 2-week, 1-month, 3-month, and 6-month posttreatment follow-up time points. The primary outcome is the WHO Disability Assessment Schedule 2.0. The secondary outcomes include alcohol use on the Timeline Follow-Back calendar, the Penn Alcohol Craving Scale, the Obsessive-Compulsive Drinking Scale, the Alcohol Craving Questionnaire, the Neurobehavioral Symptom Inventory, the PTSD Checklist for *DSM-5*, the Beck Depression Inventory-II, the Beck Anxiety Inventory, and the Mood and Anxiety Symptom Questionnaire.

**Results:**

This study protocol was approved by the institutional review board of the Edward Hines Jr Department of Veterans Affairs Hospital (19-021). This study includes a Food and Drug Administration investigational device exemption (G180292). A 6-year research plan timeline was developed, including cost and no-cost extensions due to the COVID-19 pandemic. As of June 2025, overall, 27 veterans with AUD+mTBI who had been enrolled in the study had completed the neural target identification phase and were awaiting enrollment in the RCT phase. Data collection for the RCT phase will be initiated soon and is expected to be completed by April 2026. We expect the results of this study to be available by May 2027.

**Conclusions:**

We will be able to provide preliminary evidence of the efficacy, safety, and feasibility of a novel TMS target for veterans with AUD+mTBI.

**Trial Registration:**

ClinicalTrials.gov NCT04043442; https://www.clinicaltrials.gov/study/NCT04043442

## Introduction

### Background

#### Clinical Problem

##### Alcohol Use Disorder

Alcohol use disorder (AUD) is defined by the American Psychiatric Association as impaired control over alcohol use characterized by the following symptoms: tolerance, withdrawal, relapse, craving, and impairments in social and occupational functioning [1]. Alcohol-related characteristics (ARCs) are defined for this study per these symptoms as well as AUD-specific outcomes that include but are not limited to alcohol craving, alcohol use, and AUD duration.

Alcohol is the most widely used substance by veterans [2], who have higher AUD rates than American civilians [2]. However, existing AUD treatments remain suboptimal. Although the pharmacotherapies naltrexone and acamprosate are efficacious for AUD, a recent meta-analysis indicates small effects (Hedges *g*=0.21) compared to placebo [3]. High rates of return to hazardous alcohol consumption after treatment (approximately 60%) [4] necessitate the development of more efficacious primary or adjunct interventions.

##### Co-Occurrence of AUD With Mild Traumatic Brain Injury

Among veterans with mild traumatic brain injury (mTBI), AUD rates are as high as 35% [5,6]. Both our research and that of others show that co-occurring AUD and mTBI (AUD+mTBI) may exacerbate brain impairment, exacerbate symptoms (eg, alcohol craving and cognitive dysfunction), and negatively impact rehabilitation effectiveness [7-11]. TBI severity is defined by the American Congress of Rehabilitation Medicine [12] as well as the Department of Veterans Affairs (VA) and the Department of Defense [13] based on the presence and duration of loss of consciousness, alteration of consciousness, and posttraumatic amnesia. mTBI is defined as a period of loss of consciousness <30 minutes, alteration of consciousness <24 hours, or posttraumatic amnesia <24 hours, along with a Glasgow Coma Scale score of 13-15 [12,14].

Among veterans with AUD+mTBI, the prevalence rates of co-occurring posttraumatic stress disorder (PTSD) and depression are as high as 44% and 23%, respectively [15,16]. Our work indicates that compared to veterans with AUD alone, veterans with AUD+mTBI and co-occurring mental health conditions self-report higher alcohol craving, a factor associated with relapse [11].

#### Knowledge Areas to Be Advanced Enabling Customized Treatment Development

##### Understanding the Relationships Between ARCs and Function

AUD and mTBI each negatively impact multiple domains of functioning. Individuals with AUD report high levels of functional disability in terms of impaired cognition, participation, and work and home life [17]; for example, AUD duration (an ARC) is negatively associated with self-reported global disability [17]. People with mTBI also report high levels of disability [18]. The functional domains of cognition, self-care, relationships, life activities, and participation in society are particularly impacted after mTBI [18].

Among veterans with AUD+mTBI, functional impairments are exacerbated. Co-occurring mTBI and substance use disorders (SUDs) worsen functional disability among veterans [19].

##### Understanding Unique AUD+mTBI Brain State Relative to Function

AUD and mTBI each affect overlapping and unique neural pathways. Advanced neuroimaging can be used to identify dysfunctional neural networks [20]. Research using resting state functional magnetic resonance imaging (rs-fMRI), for example, demonstrates that most networks supporting task-based function communicate between brain regions via synchronized activity at rest [21]. In addition, rs-fMRI within established networks is associated with biological changes and pathological behavioral outcomes associated with neuropsychiatric conditions [22], suggesting that the condition of rs-fMRI reflects the ability to function. Similarly, white matter integrity assessed through diffusion tensor imaging (DTI) and gray matter volume are substantially low in some neuropsychiatric conditions, indicating that these measures are relevant to healthy brain and behavioral function [23,24]. Understanding the unique AUD+mTBI brain state is vital to understanding which neural substrates, when targeted with neuromodulation, are likely to optimize treatment responsiveness, replacing or complementing current pharmacotherapies that, when administered alone, yield suboptimal treatment outcomes.

##### Neuroimaging Techniques for Customized Transcranial Magnetic Stimulation Target Site Determination

We used multimodal neuroimaging metrics to inform the selection of our repetitive transcranial magnetic stimulation (rTMS) neural target. To understand what is unknown for AUD+mTBI, we examined published neuroimaging literature characterizing the circuitry implicated in AUD and mTBI each alone [8]. While we know that some brain regions and networks implicated in AUD alone overlap with mTBI alone and that others are unique to each condition [8], we do not know how co-occurring mTBI affects the AUD brain state to influence function.

Neurophysiological changes within the whole brain, specific networks, and regions of interest can be assessed using multimodal techniques to derive neuroimaging metrics, including (1) task-based fMRI, (2) rs-fMRI, (3) DTI of white matter integrity, and (4) gray matter density. First, task-based fMRI was used to assess brain activation in response to visual alcohol cues to identify brain regions that have increased or decreased responsiveness compared to veteran controls [25]. This task is clinically relevant because visual cues (eg, pictures of alcohol or people drinking in bars) are related to alcohol craving [25], which is associated with relapse [26]. Second, we used rs-fMRI to examine how brain networks function together at rest [27]. rs-fMRI is free from task-related confounds such as differing attention levels and cognitive abilities [27]. The rs-fMRI metric informed whether connectivity between cortical and subcortical brain regions is pathological in terms of hyper- or hypoconnectivity, which is important because of evidence that rTMS applied to a cortical site can have subcortical effects [20,28,29]. Thus, rs-fMRI data can be used to inform the optimal cortical rTMS target with desired subcortical effects. Third, DTI is an indirect index of the structural integrity and connectivity of white matter tracts comprised in neural networks [30]. DTI can identify structural dysfunction between cortical and subcortical structures, and these tracts can be targeted with neuromodulatory treatment. Finally, metrics of gray matter density and volume measure brain atrophy [31] and complement DTI and rs-fMRI metrics; for example, if there is a common site of atrophy, then this site would not be selected for neuromodulation, but a site that is structurally or functionally connected to the atrophic site may be selected to enable effective neural compensation.

**Figure 1:**
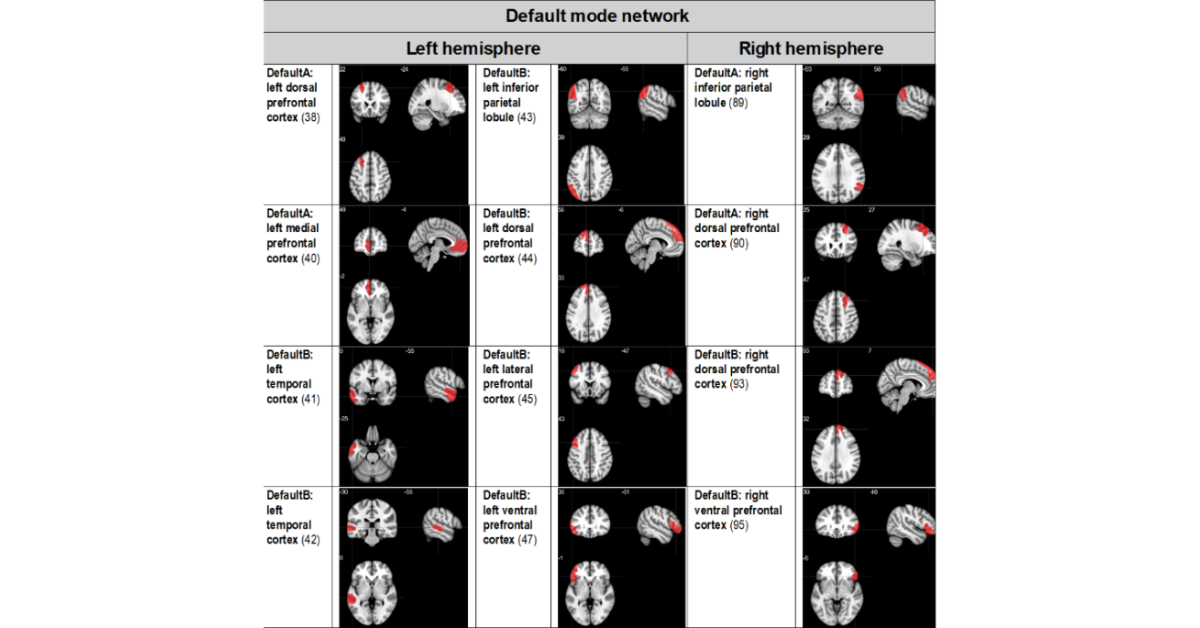
Potential cortical default mode network transcranial magnetic stimulation target treatment sites. Regions of interest are derived from the 17-network, 100-parcellation atlas based on the study by Schaefer et al [32,33], and include the default mode network, the salience and ventral attention network, and the frontoparietal control network. Regions of interest that presented surface cortical access were chosen. The numbers in parentheses indicate the Schaeffer atlas parcel number.

**Figure 2:**
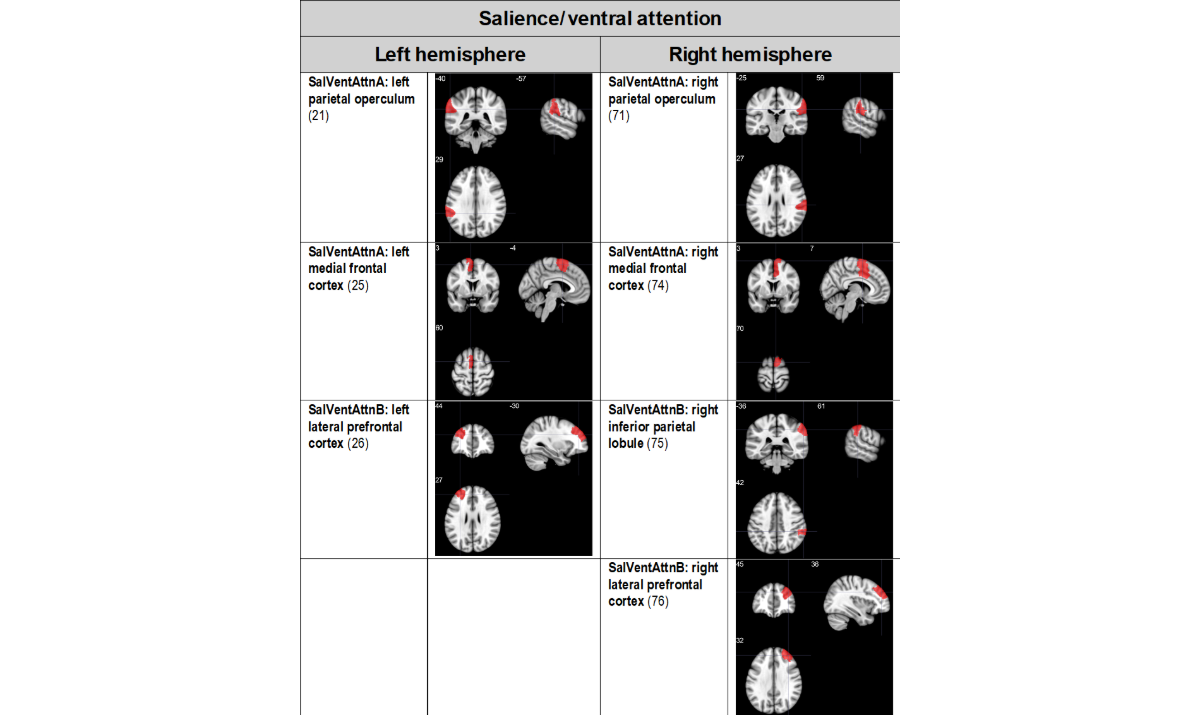
Potential salience and ventral attention network (SalVentAttn) transcranial magnetic stimulation target treatment sites. Regions of interest are derived from the 17-network, 100-parcellation atlas based on the study by Schaefer et al [32,33]. Regions of interest that presented surface cortical access were chosen. The numbers in parentheses indicate the Schaeffer atlas parcel number.

**Figure 3:**
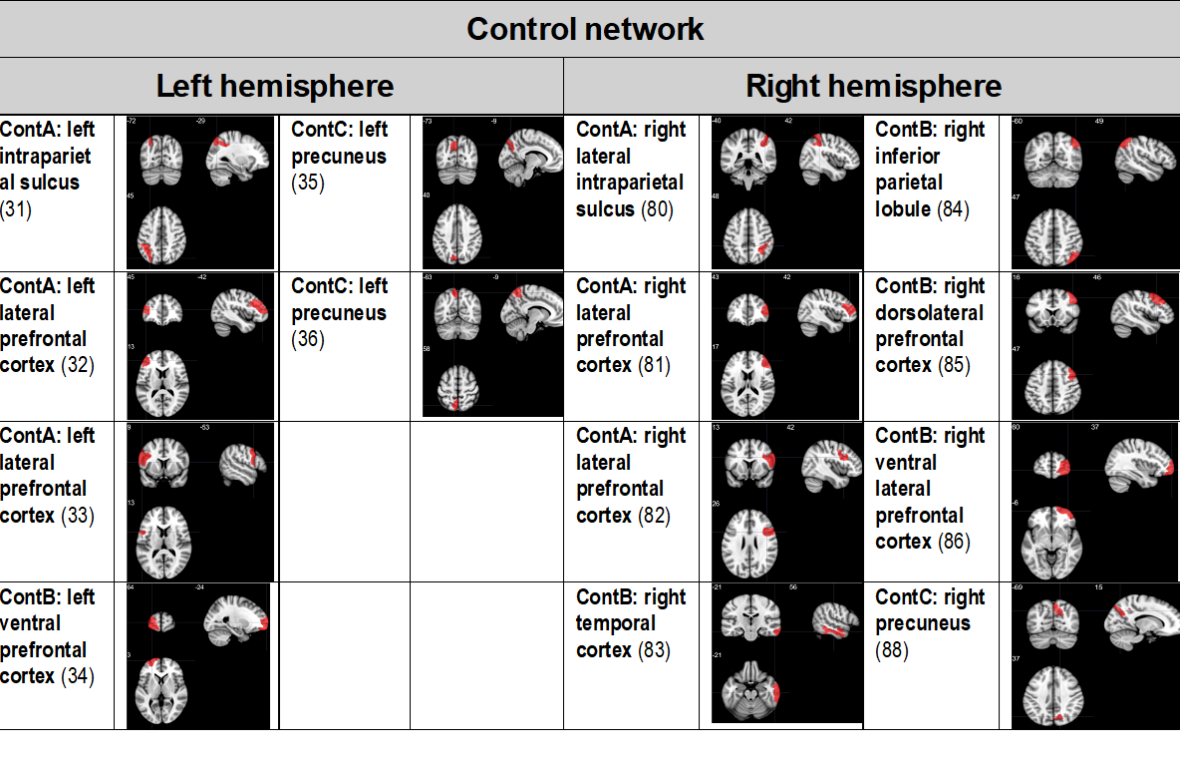
Potential frontoparietal control network (Cont) transcranial magnetic stimulation target treatment sites. Regions of interest are derived from the 17-network 100-parcellation atlas based on the study by Schaefer et al [32,33]. Regions of interest that presented surface cortical access were chosen. The numbers in parentheses indicate the Schaeffer atlas parcel number.

We focused our analysis of multimodal neuroimaging metrics on the following brain networks implicated in both AUD and mTBI [8]: the default mode network, executive control network, the frontoparietal control network, and the salience and ventral attention network. Ongoing data analyses will select the cortical region of interest from these networks as the customized neural target for rTMS, and the results will be published elsewhere (manuscript in preparation). Potential cortical TMS treatment target sites from the default mode network, the salience and ventral attention network, the executive control network, and the frontoparietal control network are summarized in Figures 1- 3, respectively. Target sites were evaluated in a prior phase of the project, and a manuscript is in preparation for publication elsewhere.

##### Rationale: Customized AUD+mTBI Neuromodulation

rTMS may provide a complementary or alternative treatment because it is noninvasive. In addition, neural targets (ie, stimulation sites) and parameters (eg, excitatory or inhibitory) can be customized to unique needs, and preliminary evidence indicates that rTMS attenuates the symptoms and functional compromise of AUD [34-37] and mTBI independently [38-40]. A review of rTMS as a treatment for AUD alone and mTBI alone further supports our contention that rTMS is a strong candidate as an alternative treatment for AUD+mTBI [8]. Published clinical studies examining rTMS for AUD alone indicate that rTMS induces a moderate effect size for reducing alcohol craving (η^2^=0.40). The largest and most promising trial used high-frequency (10 Hz), excitatory (110% motor threshold [MT]) rTMS to the right dorsolateral prefrontal cortex (DLPFC) [34]. Notably, the rTMS effect is an improvement on existing pharmacotherapies (Hedges *g*=0.21) [3]. Furthermore, the same rTMS protocol to the left or right DLPFC significantly (*P*<.05) reduced alcohol craving for people with AUD alone, with no effect size differences between sites [41]. Among people with AUD, recent findings indicate that an inhibitory patterned type of rTMS, continuous theta burst stimulation (inhibitory), to the left frontal pole in a single session resulted in reduced alcohol cue–induced brain activation within reward circuitry. An effect on alcohol craving was not expected because only a single treatment session was provided, highlighting the need for research on multisession rTMS with AUD+mTBI. In addition, a pilot randomized controlled trial (RCT) examining the utility of intermittent theta burst stimulation (iTBS; excitatory) administered to the left DLPFC for AUD among veterans was recently published. This pilot RCT found that veterans with AUD who received active iTBS were less likely to relapse and experienced greater reductions in anhedonia compared to those who received sham iTBS [42].

There are several case studies on rTMS for TBI [8,43] as well as 8 RCTs of rTMS as a treatment for postconcussive or posttraumatic depression, headache, and chronic central pain among individuals with mTBI, all with modest sample sizes (n≤30) [44]. These trials indicate that high-frequency (10 Hz) and excitatory (100%-120% MT) rTMS is efficacious for mTBI using various stimulation sites, improving mTBI symptoms (left DLPFC) [45], pain and health-related quality of life (individualized motor cortex) [40] as well as headache (although 70% MT) [46], and depression [39] (left DLPFC). However, low-frequency (1 Hz) right DLPFC rTMS was used in 2 RCTs with improved postconcussive depression symptoms as well as working memory and executive function in the study (n=13) by Lee and Kim [47] but without significant improvement in depression symptom outcomes in the study (n=30) by Rao et al [48]. In addition, 2 RCTs provided bilateral rTMS with high frequency (10 Hz) to the left DLPFC and low frequency (1 Hz) to the right DLPFC with improved depression symptoms among the active versus sham rTMS groups in the study (n=15) by Siddiqi et al [49] but without improvement in the study (N=21) by Hoy et al [50]. Of note, the study by Siddiqi et al [49] used individualized rs-fMRI data to inform rTMS targeting and a sham coil, while the study by Hoy et al [50] used a treatment coil angled 45° off the head as the sham condition.

A gap in this literature is that veterans were included in only 1 TMS AUD study [42] and only 1 TMS mTBI study. In addition, most of these rTMS trials used the left or right DLPFC for rTMS provided at 10 Hz (90%-110% MT), which also has demonstrated efficacy with depression [51]. The national VA clinical TMS program recently published findings demonstrating that among its sample of 770 veterans who received TMS for depression, 50.7% reported a prior mTBI (ie, a period of loss of consciousness <30 min and posttraumatic amnesia <24 hours, along with no structural imaging findings). Most patients received 10 Hz rTMS to the left DLPFC (120% MT; 3000 pulses per session for 30 sessions). mTBI status did not predict rTMS-related changes in depression symptom outcomes, and there was no relationship between the number of mTBIs and TMS-related depression remission rates [52]. However, mTBI status was related to a modest rTMS-related improvement in PTSD symptoms [52]. Thus, these naturalistic study results collected among a large sample of veterans provide evidence to support the safety of high-frequency rTMS among veterans with co-occurring mTBI.

However, the use of neuroimaging evidence to select neural targets customized for AUD+mTBI has not been examined. High-frequency stimulation has been efficacious for improving mTBI [38], depression [51], and PTSD symptoms [8]. As we seek to also treat AUD, we will use the parameters demonstrated by Mishra et al [34] as efficacious for reducing alcohol craving (an ARC). Another AUD study by Mishra et al [41] involved the provision of 10 daily sessions (every weekday for 2 wk) of high-frequency (10 Hz) rTMS to the left DLPFC at an intensity of 110% MT. As described previously, patterned types of rTMS, such as TBS, have been examined in PTSD and AUD. A single case study using iTBS as a potential treatment for TBI has been reported [53]. The study details the case of a man aged 25 years with TBI who underwent 3 weeks of cerebellar iTBS combined with rehabilitation. This patient experienced improvements in balance performance, motor recovery, step length, and walking speed after iTBS [53]. Importantly, this report suggests that iTBS can be used among individuals with TBI. However, given the larger body of evidence for high-frequency rTMS for AUD treatment efficacy [8] and the more established safety profile [54], high-frequency (10 Hz) rTMS is a logical first step for treating individuals with AUD+mTBI, a population with elevated risk of seizures associated with potential alcohol withdrawal.

Our hypothesis—that the provision of rTMS to a customized neural target identified using multimodal neuroimaging metrics for AUD+mTBI will optimize rTMS effects on functional disability—is based, in part, on the use of rs-fMRI among healthy controls and people with depression. Previous research demonstrated that it is possible to identify a cortical stimulation site using rs-fMRI to modulate a subcortical target using rTMS [20]. Wang et al [20] found that when rTMS was applied to the posterior parietal cortex, the hippocampus was modulated, and both rs-fMRI and associative memory were strengthened. rs-fMRI was also used to develop neuroimaging metrics for people with depression by depression subtypes [55]. Structural MRI was used to determine that specific rTMS stimulation sites within the left DLPFC (ie, lateral or anterior) were related to better depression treatment responsiveness [56]. DTI data were used to define the rTMS treatment site within the injured spinothalamic tract to improve chronic pain among people with mTBI [40].

### Objectives

This study aims to (1) test the preliminary efficacy of high-frequency rTMS provided to a customized neural target to treat functional disability among veterans with AUD+mTBI and (2) assess the sustainability of rTMS effects on functional disability among veterans with AUD+mTBI.

## Methods

### Study Design

This is a single-blind RCT involving veterans with AUD+mTBI randomly assigned to (1) a customized TMS target site of stimulation or (2) standard clinical left DLPFC. The protocol was developed according to the SPIRIT (Standard Protocol Items: Recommendations for Interventional Trials) statement [57].

A 6-year research plan timeline was developed, including cost and no-cost extensions due to the COVID-19 pandemic (Table 1).

**Table 1:** Research plan timeline.

Tasks	Year 1	Year 2	Year 3	Year 4	Year 5	Year 6
Study start-up	✓					
COVID-19 study administrative hold	✓^a^	✓^a^				
Customized TMS^b^ target identification phase: participant procedures		✓	✓	✓	✓	
Neuroimaging processing			✓	✓	✓	
TMS RCT^c^ treatment phase procedures					✓	✓
Data analysis				✓	✓	✓
Dissemination						✓

^a^From March 2020 to February 2021.

^b^TMS: transcranial magnetic stimulation.

^c^RCT: randomized controlled trial.

### Ethical Considerations

#### Human Participant Ethics Review Approvals

This study involves human participant research and adheres to appropriate ethics review approvals. This study protocol was approved by the institutional review board (IRB) of the Edward Hines Jr VA Hospital in Hines, Illinois, United States (19-021), on August 7, 2019, and was prospectively registered on ClinicalTrials.gov (NCT04043442). This study includes a Food and Drug Administration (FDA) investigational device exemption (G180292). The research study staff will be responsible for communicating any protocol amendments to the IRB and any investigational device exemption supplements to the FDA, as appropriate. Significant amendments will be submitted to the study sponsor (VA Office of Rehabilitation Research Development and Translation) as a project modification for prior review and approval.

#### Informed Consent

All study participants will provide informed consent before completing research procedures. Authorized clinical researchers will meet eligible participants at the designated entrance, closest in proximity to a private room where the study visit is to take place. The researcher will provide all documentation, including the study consent form and all assessments that the participant will need to complete.

All participants will be able to make their own decisions regarding research participation. They will be encouraged to discuss study participation with a family member or friend before signing the consent form. The participant will not be openly encouraged to participate in the research or told that there is any expected benefit from the experimental interventions during participation. Participants will be given the option to opt out of consenting or research procedures at any time without loss or penalty. Research participants will have access to research staff to assist with any questions or concerns until understanding is achieved to the judgment of the individual asking the question. If a participant refuses participation, no further contact will be made. If signed informed consent forms are completed, a note will be made in the Edward Hines Jr VA Hospital electronic medical record (EMR), and the original consent form will be kept in a locked cabinet in a locked research office behind swipe-access doors. Copies of the signed consent forms will be provided to the Edward Hines Jr VA Hospital IRB office. In addition, a copy will be provided to the participant.

#### Privacy and Confidentiality

All hard copies of assessments will include only a unique participant ID number and the date the assessment was completed to maintain deidentification. The only documents containing identifiable information will be the demographics questionnaire and the contact information form. The demographic questionnaire will be kept in a locked filing cabinet within the locked office of the study principal investigator (PI), AAH. Each participant’s name and contact information will be linked to the unique study ID number and saved electronically on a secure VA server containing the cross-walk file at the Edward Hines Jr VA Hospital. This secure cross-walk file is the only place where identifying information (eg, name, address, telephone number, date of birth, and social security number) is associated with unique participant ID numbers. To further protect participant confidentiality, the PI will have control over which research team members are allowed access to the cross-walk file.

#### Compensation Details

Participants will receive US $200 for completing all rTMS treatments (sham and active). They will receive up to US $75 for completing all 3 follow-up visits (US $25 per visit). Therefore, at the end of research participation, participants are eligible to receive up to US $275. This compensation is intended to facilitate participation without adding undue influence.

### Setting

Treatment-seeking veterans from the Edward Hines Jr VA Hospital will be included. All research procedures will take place at this hospital.

### Participants

We will target the enrollment of 46 veterans with AUD+mTBI. Complete study eligibility criteria are presented in Textbox 1. Veterans aged 22 to 70 years without a history of moderate to severe TBI, neurodegenerative disorders, psychotic spectrum disorders, MRI contraindications, intellectual disability (Wechsler Test of Adult Reading predicted full-scale IQ score <70) [58], or the use of illicit substances in the past 30 days (except for cannabis) will be eligible. The lower age limit was set as 22 years because the FDA considers individuals aged ≥22 years as adults. The upper age limit of 70 years was selected to eliminate potential confounds related to neural aging processes and the potential for neurodegenerative disease onset. Veterans will meet *Diagnostic and Statistical Manual of Mental Disorders, Fifth Edition* (*DSM-5*) [1], criteria for AUD per the Structured Clinical Interview for *DSM-5* [59] and the symptom attribution and classification algorithm (SACA) classification criteria for mTBI. This algorithm, developed by our team [60], is systematic and consistent with the American Congress of Rehabilitation Medicine [12], VA, and Department of Defense mTBI criteria [13].

**Participant eligibility criteria.**

**Inclusion criteria**

- Aged 22 to 70 years
- Can read and speak English
- *Diagnostic and Statistical Manual of Mental Disorders, Fifth Edition* (*DSM-5*), criteria for alcohol use disorder symptom attribution and classification algorithm (SACA), criteria for mild traumatic brain injury (mTBI; without requirement of clinical neuropsychological impairment)

**Exclusion criteria**

- History of moderate to severe TBI
- Within 1 year of mTBI
- History of nontraumatic neuroinjury (eg stroke, neurosurgery, or hemorrhage)
- Neurodegenerative disease (eg, Alzheimer disease, Parkinson disease, or multiple sclerosis)
- Has an active, unstable health condition (ie, decompensated heart failure or recent severe heart attack)
- Within 12 weeks of major surgery
- History of psychosis or current psychosis not attributable to an external cause (eg, illicit drug use)
- Endorses active suicidal ideation with intent and plan (these participants will be brought to the emergency department for emergent psychiatric care)
- Intellectual disability (Wechsler Test of Adult Reading predicted full-scale IQ score <70) [58]
- Is pregnant or nursing
- Use of benzodiazepines, opiates, cocaine, or amphetamines in the past 30 days
- Meets *DSM-5* criteria for moderate to severe cannabis use disorder
- Contraindications to magnetic resonance imaging (eg, claustrophobia, ferromagnetic metal in eyes or face, congestive heart failure, implanted cardiac pacemaker or defibrillator, cochlear implant, or nerve stimulator)
- Contraindications to intermittent theta burst stimulation and transcranial magnetic stimulation (eg, epilepsy or taking medications that elevate the seizure threshold)
- Meets SACA criteria for “questionable validity” of performance effort and symptom reporting

To enhance generalizability, veterans will be included irrespective of combat and deployment status. As cannabis use is prevalent among veterans, including those with AUD, cannabis users and those with mild cannabis use disorder (per *DSM-5*) [1,59] will not be excluded; given that legal cannabis purchase and consumption are likely to increase across the United States, the inclusion of cannabis users will increase the generalizability of the findings. As daily cannabis use and cannabis dependence have significant relationships with ARCs, veterans with moderate to severe cannabis use disorder will be excluded. Depression, anxiety, and PTSD commonly co-occur among veterans [61]. As this is the population we seek to generalize our study findings to, participants who meet *DSM-5* [1] criteria for depression, anxiety, or PTSD will not be excluded. To maintain high-quality data, any participant meeting criteria for questionably valid symptom profiles (ie, effort and symptom validity) will be excluded. The forced choice component of the California Verbal Learning Test–Second Edition will be used, with a cutoff score of 15, as a measure of effort performance [62]. The Minnesota Multiphasic Personality Inventory-2–Restructured Form [63] will be used to identify atypical symptom reporting based on the following criteria: infrequency scale T score ≥107, infrequency-psychopathology scale T score ≥85, true response inconsistency scale T score ≥80, and variable response inconsistency scale T score ≥80 [60].

There are no study participation limitations based on gender, race, or ethnicity.

### Recruitment

To recruit a study sample representative of veterans with AUD+mTBI, we will target treatment-seeking veterans from the Edward Hines Jr VA Hospital, which houses a residential SUD treatment program, an outpatient SUD treatment program, and a TBI and polytrauma program (ie, a VA polytrauma network site).

We will identify research candidates at this VA hospital using the national inpatient and outpatient files available through the VA Informatics and Computing Infrastructure. We will request access to data housed in the Corporate Data Warehouse, including production domains and vital status files (such as Beneficiary Identification and Record Locator Subsystem), as well as Compensation and Pension Record Interchange (CAPRI) and Veterans Health Information Systems and Technology Architecture (VistA), with real social security number identifiers located in VA Informatics and Computing Infrastructure using the Data Access Request Tracker system. Once this access is approved, we will search the database for the past 10 years using appropriate *International Classification of Diseases, Ninth Revision* and *International Classification of Diseases, Tenth Revision, Clinical Modification* codes reflecting study inclusion criteria. These files will be accessed for the duration of the study.

After identification, veterans’ medical records will be reviewed for eligibility, and eligible veterans will be mailed an informational letter. The study team will follow up with a telephone call 1 week after the letter is mailed to inquire whether the veteran is interested, and, if so, screen further for eligibility. The study team will also distribute IRB-approved flyers and present the study at relevant staff meetings for VA providers. VA providers will also be given IRB-approved flyers and encouraged to give them to veterans who may be a good fit for the study. The flyers will contain a study contact telephone number so that veterans can call the study team if they are interested in participating.

The veterans with AUD+mTBI who completed the neural TMS site determination phase will be called to schedule research procedures for the rTMS treatment phase. There will be a gap of 1 month to 4 years between the completion of study phases, and quarterly check-in calls will be completed to maintain study contact with participants.

### Data Collection and Measures

#### Overview

**Figure 4:**
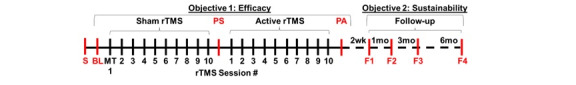
Study timeline. BL: baseline visit; F1: follow-up time point visit occurring 2 weeks after the last active repetitive transcranial magnetic stimulation (rTMS) session; F2: follow-up time point visit occurring 1 month after the last active rTMS session; F3: follow-up time point visit occurring 3 months after the last active rTMS session; F4: follow-up time point visit occurring 6 months after the last active rTMS session; MT: motor threshold visit; PA: post–active rTMS visit; PS: post–sham rTMS visit; S: screening visit.

Data collection occurred across multiple study time points, beginning with screening and baseline visits and continuing through posttreatment and follow-up visits. The full study timeline is presented in Figure 4. An overview of the assessments collected at each time point is provided in Table 2.

**Table 2:** Study assessments, grouped by time point collected.

Time points and assessments administered			Assessment purpose
**Telephone screening**			
	AUDIT-C^a^	Screen for AUD^b^	
	OSU TBI-ID^c^	Screen for TBI^d^	
	TMS^e^ safety form	Screen for TMS safety	
	Medication inventory form	Screen for TMS safety	
**Screening visit**			
	SCID-5^f^ AUD module	Diagnosis and duration of AUD	
	Structured diagnostic interview [60] with the NSI^g^ [64]	mTBI^h^ classification and symptoms	
	CIWA-Ar^i^	Determine acute alcohol withdrawal status	
	TLFB^j^ alcohol calendar, past year	Determine alcohol consumption history	
	C-SSRS^k^	Determine acute suicidality	
**Baseline, post–sham repetitive TMS, post–active repetitive TMS, 2-wk, 1-mo, 3-mo, and 6-mo follow-up visits**			
	WHODAS 2.0^l^	Self-reported functional disability (primary outcome)	
	PACS^m^	Self-reported alcohol craving (secondary outcome)	
	OCDS^n^	Self-reported alcohol craving (secondary outcome)	
	ACQ-NOW^o^	Self-reported alcohol craving (secondary outcome)	
	NSI	Self-reported mTBI symptoms (secondary outcome)	
	PCL-5^p^	Self-reported PTSD^q^ symptoms (secondary outcome)	
	BDI-II^r^	Self-reported depression symptoms (secondary outcome)	
	BAI^s^	Self-reported anxiety symptoms (secondary outcome)	
	MASQ^t^	Self-reported mood and anxiety symptoms (secondary outcome)	
	FTND^u^	Self-reported nicotine use symptoms (secondary outcome)	
	C-SSRS	Determine acute suicidality	

^a^AUDIT-C: Alcohol Use Disorder Identification Test–Consumption Questions.

^b^AUD: alcohol use disorder.

^c^OSU TBI-ID: Ohio State University Traumatic Brain Injury Identification Method.

^d^TBI: traumatic brain injury.

^e^TMS: transcranial magnetic stimulation.

^f^SCID-5: Structured Clinical Interview for *DSM-5*.

^g^NSI: Neurobehavioral Symptom Inventory.

^h^mTBI: mild traumatic brain injury.

^i^CIWA-Ar: Clinical Institute Withdrawal Assessment for Alcohol, Revised.

^j^TLFB: Timeline Follow-Back.

^k^C-SSRS: Columbia–Suicide Severity Rating Scale.

^l^WHODAS 2.0: World Health Organization Disability Assessment Schedule.

^m^PACS: Penn Alcohol Craving Scale.

^n^OCDS: Obsessive-Compulsive Drinking Scale.

^o^ACQ-NOW: Alcohol Craving Questionnaire.

^p^PCL-5: PTSD Checklist for *DSM-5*.

^q^PTSD: posttraumatic stress disorder.

^r^BDI-II: Beck Depression Inventory-II.

^s^BAI: Beck Anxiety Inventory.

^t^MASQ: Mood and Anxiety Symptom Questionnaire.

^u^FTND: Fagerström Test for Nicotine Dependence.

#### Screening

Veterans identified as research candidates will complete an initial telephone screening for the following: (1) probable AUD, using the Alcohol Use Disorder Identification Test–Consumption Questions (AUDIT-C) [65]; (2) probable mTBI, using the Ohio State University Traumatic Brain Injury Identification Method [66]; (3) TMS safety, using our TMS safety form; and (4) self-reported age as well as the ability to read and speak English. Age will be verified in the EMR (ie, Computerized Patient Record System or Compensation and Pension Record Interchange). The AUDIT-C is a 3-item, self-report measure of alcohol use; scores range from 0 to 12, with a score of ≥4 for men and ≥3 for women indicating hazardous drinking and probable AUD. A list of prescribed and over-the counter medications and the length of time the participant has been on each medication will also be collected over the telephone. Current medications will be cross-referenced with study eligibility criteria and the EMR to identify any antiepileptics as well as any medications known to lower seizure threshold. The EMR will also be reviewed for study eligibility criteria. If review findings indicate possible contraindications to rTMS or MRI related to a metal implant, attempts will be made to identify the model and manufacturer of the implant to determine whether it is safe to expose the implant to a strong magnet. Manufacturer recommendations regarding safety will be followed. If potential participants meet all eligibility criteria, they will be scheduled for the first research visit. They will be mailed a copy of the informed consent form to review, along with a reminder of the appointment date and time.

Veterans meeting the initial screening criteria for the AUD+mTBI group will screen positive for the AUDIT-C and report at least 1 lifetime mTBI on the Ohio State University Traumatic Brain Injury Identification Method.

All veterans aged 22 to 70 years without MRI safety contraindications who can read and speak English will be eligible for enrollment and diagnostic screening as outlined in the following paragraphs. Veterans meeting these criteria will complete behavioral assessments during visit 1 that will be used to confirm study eligibility.

We will complete the Timeline Follow-Back alcohol calendar for the past year, meeting with a study physician to review current medications, recent drinking history, the Columbia–Suicide Severity Rating Scale (C-SSRS), and the Clinical Institute Withdrawal Assessment for Alcohol, Revised.

Abstinence from alcohol and illicit substances will be requested for each in-person visit and will be confirmed with a urine alcohol and urine drug screen and breathalyzer or urine or saliva test for alcohol following evolving hospital COVID-19 guidelines. If veterans opt for an in-person visit 1, the urine drug screen will be completed by a VA clinical laboratory using a manual requisition form with the veteran’s unique participant ID number. All subsequent urine drug screens will be conducted by research staff using a urine test kit. The results from the drug and alcohol screens will not be entered into the veteran’s medical record; they are for research purposes only. Urine specimens will not be stored. Participants will also complete an acute cannabis intoxication scale [67] that includes four items: (1) increased resting heart rate (>100 beats per min), (2) congestion of the conjunctival blood vessels (red eyes), (3) slowed speech, and (4) giddiness. Any participant testing positive for alcohol, drugs, or cannabis intoxication will be asked to reschedule research procedures, and safe transportation will be arranged (eg, taxi), or participants will be escorted to the emergency department (ED) or mental health intake, as appropriate.

#### Baseline, Post–Sham rTMS, Post–Active rTMS, 2-Week, 1-Month, 3-Month, and 6-Month Follow-Up Visits

All veterans will complete the primary outcome measure: the WHO Disability Assessment Schedule (WHODAS 2.0), a self-report of functional disability. The following ARCs will also be collected as secondary outcomes: the Penn Alcohol Craving Scale, the Obsessive-Compulsive Drinking Scale, the Timeline Follow-Back (including the number of days between baseline and screening visits), and the Alcohol Craving Questionnaire. The following additional secondary outcomes will also be assessed: the Neurobehavioral Symptom Inventory, the PTSD Checklist for *DSM-5*, the Beck Depression Inventory-II, the Beck Anxiety Inventory, and the Mood and Anxiety Symptom Questionnaire. The C-SSRS will be repeated at baseline, after both sham and active rTMS treatment session 5, and at post–sham rTMS and post–active rTMS visits. We will also assess self-reported smoking status (ie, current, former, or never smoker, with never smoker defined as having smoked <100 cigarettes in a lifetime) and the Fagerström Test for Nicotine Dependence.

Finally, all female participants will complete a urine pregnancy test using a test kit at baseline and post–sham rTMS time points.

We will be using the VA REDCap (Research Electronic Data Capture; Vanderbilt University) secure web application for data collection and data entry. Data will be checked for completeness at the time of assessment and again at the time of data entry. Reasons for missingness will be documented.

### rTMS Intervention

#### Randomization to Stimulation Site Group

Veterans with AUD+mTBI will be randomly assigned to 1 of 2 stimulation site groups: a customized site or left DLPFC. SAS 9.4 (SAS Institute Inc) will be used to develop the randomization protocol for the veterans, ensuring an equal number of participants—assigned sequentially—in each of the 2 groups. An electroencephalography (EEG) cap along with the coordinate Beam F3 method and neuronavigation will be used for each participant to maintain the single-blind design. The randomization scheme will be provided by the biostatistician to a single unblinded administrator (who will not complete any research procedures or analyses). This administrator will inform the rTMS provider of the patient’s assigned site (left DLPFC or a customized site) for rTMS treatment.

The customized site will be one of the sites depicted in Figures 1- 3- as determined by ongoing analyses.

#### MT and Brain Mapping

Each participant’s T1-weighted MRI scan will be loaded into a Localite TMS Navigator system. A MagVenture C-B60 coil will be used to deliver single-pulse TMS to the left motor cortex to identify the abductor pollicis brevis (ie, thumb muscle) coordinates. Resting MT will be guided by adaptive parameter estimation by sequential testing procedures [68] using a web-based tool [69] and defined as the lowest stimulus intensity necessary to produce motor evoked potentials of peak-to-peak amplitude of ≥50 µV in 5 of 10 trials. Motor evoked potentials of the abductor pollicis brevis will be recorded using surface electrodes on the right thumb. MT may be repeated as needed with changes in medication regimens, illness, and so on. The left DLPFC will be mapped using the standard EEG cap and coordinate Beam F3 method [70]. The customized site will be mapped using Montreal Neurological Institute (MNI) atlas coordinates. EEG caps and neural navigation will be used at every rTMS session for blinding purposes. MT procedures will be conducted by a trained TMS administrator.

#### rTMS Parameters

rTMS will be provided at 10 Hz, 4.9 seconds per train, intertrain interval of 30 seconds, 20 total trains per session, and an intensity of 110% MT [41,51,54]. Each patient will receive 1000 pulses per day. The sham and active rTMS sessions will each be provided daily for 2 weeks (once per d, 5 d per wk; Figure 4). If a date is missed, it will be made up as soon as possible at the end of treatment. The rates of missed sessions and durations between sessions will be recorded and used as potential covariates in analyses. rTMS procedures will be conducted by a trained TMS administrator.

#### Single-Blind Design

A single-blind design will be maintained. Veterans will know that they will be receiving sham and active rTMS but will not know when they are receiving which treatment. The consent form will specify that they will be blinded to treatment order. The biostatistician will be blinded to group. Researchers will be unblinded to treatment order because all participants receive sham rTMS first and active rTMS second. This is necessary because the “washout” period of rTMS effects is unknown, and objective 2 examines sustainability.

#### rTMS Device and Blinding Procedures

Sham and active rTMS will be delivered with the MagVenture MagPro X100 with MagOption stimulator and MagVenture Cool B65 A/P (active or placebo [aka sham]) coil, which can be switched to active or placebo. The coil is identical visually for both the sham or placebo and active conditions. During rTMS, veterans and researchers will wear headphones connected to the placebo noise generator so that they hear active TMS for both conditions, thereby maintaining the single-blind design. For sham rTMS, the coil will be switched to placebo or sham where a sensation is produced via surface electrodes placed on the forehead that mimics the sensation produced by the active coil. Thus, the sham rTMS looks, sounds, and feels similar to active rTMS.

#### Safety Measures

All investigators and research team members who will be providing the experimental treatment will undergo mandatory training on the use of the MagVenture device and the provision of active and sham rTMS.

In addition, to monitor for discomfort or pain, participants will be asked, after each rTMS session, to rate their pain. They will characterize the sensation by describing it as painful, tingling, sharp, piercing, tugging, pinching, or intolerable and rate the degree of the sensation on a scale ranging from 0 100 (100 being the worst pain you have experienced).

The rTMS treatment day safety monitoring plan and log will also be completed for each session. Each item in this log specifies whether it will be completed before, during, or after the rTMS session. The rTMS treatment day safety monitoring plan and log contains a customized severity indicator scale. For each safety variable specified in the rTMS treatment day safety monitoring plan and log, change from baseline is rated according to severity; and for each severity rating, there is a specified medical response to be followed. The ratings are on a scale ranging from 1 to 5, with a higher number indicating more deleterious change. This scale rates changes from baseline in vital signs (temperature, blood pressure, heart rate, and oxygen saturation levels), fatigue, tinnitus, sleep, dizziness, nausea, vomiting, confusion, seizure, syncope (fainting), headache, neck pain, skin integrity of the scalp, substance use, and mental health symptoms. This scale will be completed for each TMS session.

Participants will be clinically monitored for seizure by trained research staff. In the highly unlikely event that a seizure occurs, the following response plan will be followed:

1. A research team member will activate the emergency code for the Edward Hines Jr VA Hospital rapid response team (RRT).
2. Trained research staff will assess the participant’s airway, breathing, and circulation.
3. Upon arrival, the RRT will take over; assess; and treat the participant, if needed.
4. The RRT will transport the participant to the Edward Hines Jr VA Hospital ED; for participants treated at a local VA ED, the PI will contact the participant within a week and encourage them to follow up with their primary care physician, in accordance with local VA policy, which recommends that patients treated in the ED should follow up with their primary care physician within 2 weeks.
5. Any seizure will result in stopping treatment and withdrawal from the study.

An adverse event log will also be completed as necessary. In addition, an adverse event tracking log has been developed for this project. Both logs are integrated into the safety monitoring procedures of this study. All serious and nonserious adverse events will be documented in the adverse event tracking log.

All female participants will complete pregnancy tests. If a pregnancy test is positive, the participant will be discontinued from the study.

To ensure that participant medications remain stable during study participation, participants will be asked before each TMS session whether their medications have changed. In addition, medication lists will be reviewed once per week with the participant to monitor for any changes.

Any C-SSRS score of >3 will prompt a VA comprehensive suicide risk evaluation. If a participant scores >3 on the C-SSRS at any time, the VA suicide prevention team will be alerted, and either a member of the team or a trained member of the research team will complete the comprehensive suicide risk evaluation with the participant, as well as develop a suicide safety plan (if one has not been created by the participant within the past year). This information will be urgently shared with the veteran’s current mental health providers, if applicable. If the participant does not have current mental health providers, they will be offered an appointment with the mental health intake team or brought to the ED, as appropriate.

Participants will complete the Beck Depression Inventory-II; item 9 specifically assesses suicidal ideation, plan, and intent on an increasing point value scale (0-3; Table 3). To monitor for changes in suicidality, item 9 from the BDI II will be administered every week in accordance with our data safety monitoring sheet.

**Table 3:** Beck Depression Inventory-II item 9: suicidal thoughts or wishes.

Scores	Responses
0	I don’t have any thoughts of killing myself.
1	I have thoughts of killing myself, but I would not carry them out.
2	I would like to kill myself.
3	I would kill myself if I had the chance.

Participants positively endorsing ≥2 indicators (more than transient ideation with possible plan and intent) will be queried about their response before proceeding with the screening. The assessor will be trained in risk assessment, which will include a review of the frequency and severity of suicidal ideation, a discussion of past and present planning behaviors, and current intent to act on ideation and planning.

A data safety monitoring board was not required by our study sponsor because this is a single-site pilot study.

#### Research-Related Injuries

Per the Code of Federal Regulations (38 CFR 17.85), the VA will provide necessary medical treatment to research participants if they are injured by participation in this research project approved by the research and development committee and conducted under the supervision of 1 or more VA employees. Except in limited circumstances, this care will be provided at the Edward Hines Jr VA Hospital. This requirement does not apply to treatment for injuries that result from a participant’s noncompliance with study procedures. The VA does not normally provide any other form of compensation for injury. The VA has not been released from liability for negligence. Participation in this research may involve risks that are currently unforeseeable.

### Statistical Analysis

#### Sample Size Justification

Our sample size was based on a power calculation for the neural TMS target identification phase of the project using a moderate effect size of 0.65 to achieve 80% power while maintaining the type I error rate at 5%. This effect size is consistent with the WHODAS 2.0 effect sizes reported in a sample of individuals with chronic conditions (0.30-0.70) [71] and a general treatment population (0.44-1.38) [72]. After accounting for an attrition rate of 20%, at least 46 participants with AUD+mTBI will be needed for the study. Thus, we hope to enroll up to 46 participants in this pilot rTMS RCT phase. As the rTMS RCT phase is a pilot study, it is not powered, but data will inform future power calculations for larger-scale studies.

#### Data Preparation

Deidentified data will be cleaned, and appropriate missing data imputation techniques will be used [73]. Multicollinearity among ARCs will be tested; highly correlated characteristics (except for one) will be removed before the final test.

#### Covariates

Covariates (eg, age, combat and deployment status, cannabis use, and nicotine dependence) will be used in the model to adjust the effect of the intervention. A bivariate analysis will be performed to test the association between each covariate and the functional disability outcome. Only statistically significant covariates will be included in the regression model.

#### Confounders

Variables such as depression symptoms, PTSD symptoms, anticraving medication use, and psychotherapy hours, which influence both treatment site (ie, neural target or left DLPFC) and functional disability (WHODAS 2.0), will be considered as confounders. Given a treatment site and confounders, WHODAS 2.0 scores will be calculated. The parameters of the model will be estimated by the weighted regression approach where weights will be determined by the inverse of the probabilities.

#### Statistical Approach

#### Objective 1

A within-participant, longitudinal design will be used in which all veterans will first receive sham rTMS followed by active rTMS applied to the customized neural target and the standard FDA-approved treatment site (left DLPFC). Thus, there will be 2 groups. The primary outcome, functional disability (WHODAS 2.0), will be assessed at baseline, after sham rTMS, and after active rTMS. A mixed effects model, incorporating within-participant correlation from repeated measures, will be used to compare the difference between difference between sham (Figure 4: PS-BL) and active rTMS (Figure 4: PA-PS) treatments. We will compare the aforementioned difference in sham and active rTMS of the left DLPFC neural target or site of stimulation with the customized neural target group.

#### Objective 2

A recovery curve will be constructed based on repeated functional disability (WHODAS 2.0) measures at post–active rTMS, 2-week, 1-month, 3-month, and 6-month follow-up time points (Figure 4: PA, F1–F4). A mixed effects model (incorporating within-participant correlation from repeated measures) will be used to examine change (using the slope of the model) in WHODAS 2.0 from post–active rTMS to each follow-up time point for each group. We will examine the change in slope of the left DLPFC group compared to the customized neural target group using WHODAS 2.0 measures from post–active rTMS to each follow-up time point.

## Results

As of June 2025, a total of 27 veterans with AUD+mTBI who had been enrolled in the study had completed the neural target identification phase and were awaiting enrollment in the RCT phase. Data collection for the RCT phase will be initiated soon and is expected to be completed by April 2026. We expect the results of this study to be available by May 2026.

## Discussion

### Hypothesized Principal Findings

For objective 1, addressing preliminary rTMS efficacy, we hypothesize that veterans receiving active rTMS at the customized site will have significantly improved functional disability from before to after active rTMS compared to from before to after sham rTMS and compared to the Beam F3 left DLPFC site. This will be indicated by a significantly lower or improved WHODAS 2.0 total complex score from before to after active rTMS compared to from before to after sham rTMS and compared to the Beam F3 left DLPFC site.

For objective 2, addressing rTMS sustainability, we hypothesize that veterans with AUD+mTBI receiving active rTMS applied to the customized site will sustain improvements in functional disability 2 weeks, 1 month, 3 months, and 6 months after active rTMS and that these improvements will be sustained beyond those sustained with rTMS applied to the Beam F3 left DLPFC site.

### Comparison to Prior Work

Research on neuromodulation for a variety of neuropsychiatric conditions is rapidly growing. This study protocol is in line with cutting-edge research that has capitalized on information about functional connectivity and brain state to optimize TMS. Prior work created functional connectivity maps of specific neuropsychiatric symptoms such as PTSD, dysphoria, and anxiety [74,75]. However, to our knowledge, these maps are yet to be tested prospectively as TMS targets in large-scale TMS clinical trials.

Recent research in TMS for SUDs has focused on the salience and ventral attention network, particularly as a potential treatment target [36]. TMS targeting regions that include salience network hubs (eg, the anterior insula and dorsal anterior cingulate cortex) has demonstrated efficacy for smoking cessation [76], leading to the first FDA clearance of TMS for an SUD. There is burgeoning evidence that TMS targeting the salience and ventral attention network could also be promising for AUD [77]. In addition, the frontal pole is another novel potential TMS target for SUDs [78]. As mentioned previously, recent findings indicate that an inhibitory patterned type of rTMS, continuous theta burst stimulation (inhibitory), to the left frontal pole in a single session resulted in reduced alcohol cue–induced brain activation within reward circuitry among people with AUD [79]. Recent results demonstrated improvement for both active and sham TMS groups of frontal pole iTBS groups in reductions in cigarette consumption, cigarette craving, and tobacco withdrawal without between-group effects [80]. Ongoing clinical trials will determine the effects of frontal pole TMS versus left DLPFC TMS for AUD [81]. Thus, this study protocol will contribute to the field and is in line with cutting-edge TMS research.

### Strengths of the Study

To our knowledge, our study is the first to test the efficacy of TMS for individuals with AUD+mTBI, a population with few treatment options. Our study will be of particular significance to the neurorehabilitation field because there is still no consensus regarding the appropriate neural TMS target for SUDs or mTBI individually, let alone AUD+mTBI. Furthermore, although much progress has been made in the TMS neuroimaging field in identifying novel targets and circuits, few studies have directly tested these in comparison to the current clinical standard.

Exploring the preliminary efficacy and sustainability of rTMS effects on functioning, according to a customized AUD+mTBI neural target and the commonly used left DLPFC, is a fundamental first step toward developing and optimizing an rTMS treatment for veterans with AUD+mTBI, for whom there are currently limited treatment options.

We will start the process of developing a customized AUD+mTBI rTMS protocol that is clinically implementable. We will do this comparing the preliminary efficacy and sustainability of the empirically derived rTMS neural target used as a treatment site. Future work will validate the customized AUD+mTBI rTMS site in a larger-scale study examining optimal stimulation parameters and dosing. This line of scientific work will lead to more treatment options for attenuating functional disability among veterans with AUD+mTBI.

### Limitations of the Study

This study may have 3 limitations. First , the single-blind nature of this study could bias results but is also an important step for developing this novel intervention. Future, larger-scale studies should use a parallel-group design and randomly assign participants to sham and active rTMS groups in a double-blinded fashion. Second, the study was powered for the initial TMS target identification phase and not the RCT rTMS treatment phase. In addition, the COVID-19 pandemic significantly affected recruitment. Thus, the RCT rTMS treatment phase could be underpowered. However, the study is intended to provide *preliminary* efficacy data that will serve as foundational work for larger-scale studies. Third, the primary outcome—the WHODAS 2.0—and the secondary outcomes are self-report outcomes that are subjective in nature. However, these measures are standards in the field and encapsulate functional and symptom outcomes that we aim to improve for veterans with AUD+mTBI. Future, larger-scale TMS studies in individuals with AUD+mTBI could add rigor by adding objective measures such as alcohol sensors and clinician-rated functional outcomes that could corroborate patient self-report outcomes.

### Future Work and Dissemination Plan

The next step for AUD+mTBI rTMS treatment development will be to validate whether the customized neural target or site results are generalizable in a larger scale, multisite, randomized, placebo-controlled, double-blind clinical trial. Subsequently, future trials using the validated rTMS site can examine rTMS dosing (eg, 10 vs 20 sessions) or treatment parameters (eg, 10 Hz vs 20 Hz). In parallel, the rich data collected in this study and in future trials can be used to explore which neuroimaging metrics and ARCs are most strongly associated with improvements in functional disability after rTMS treatment. Using big data approaches such as machine learning, the markers of treatment candidacy and the predictors of treatment responsiveness can be evaluated. Once the optimal rTMS neural target, dosing, and parameters are defined and validated, a standardized rTMS protocol can be implemented clinically. This protocol would include the generation of a set of optimal MNI brain coordinates to be used as the rTMS treatment site and a complementary international 10-20 EEG electrode placement site that aligns with the MNI coordinates. If clinical sites are equipped with an rTMS neural navigation system, they can input the MNI coordinates onto the averaged structural T1-weighted MRI scan within the navigation system and then register the patient to this average brain. If clinical sites are not equipped with a navigation system, the EEG placement site can be used instead because using a specific EEG placement method (eg, Beam F3) has demonstrated reasonable approximation to MRI-guided scalp sites for the left DLPFC [82].

We plan to submit the trial results to scientific, peer-reviewed journals; there are no publication restrictions. A CONSORT (Consolidated Standards of Reporting Trials) diagram will be included in the publication. We will follow the International Committee of Medical Journal Editors guidelines for authorship, and there is no intention of using professional writers.

## Appendix

Multimedia Appendix 1

## Abbreviations

ARC: alcohol-related characteristic
AUD: alcohol use disorder
AUD+mTBI: co-occurring alcohol use disorder and mild traumatic brain injury
AUDIT-C: Alcohol Use Disorder Identification Test–Consumption Questions
C-SSRS: Columbia–Suicide Severity Rating Scale
CAPRI: Compensation and Pension Record Interchange
CONSORT: Consolidated Standards of Reporting Trials
DLPFC: dorsolateral prefrontal cortex
*DSM-5*: *Diagnostic and Statistical Manual of Mental Disorders, Fifth Edition*
DTI: diffusion tensor imaging
ED: emergency department
EEG: electroencephalography
EMR: electronic medical record
FDA: Food and Drug Administration
IRB: institutional review board
iTBS: intermittent theta burst stimulation
MNI: Montreal Neurological Institute
MT: motor threshold
mTBI: mild traumatic brain injury
PI: principal investigator
PTSD: posttraumatic stress disorder
RCT: randomized controlled trial
RRT: rapid response team
rs-fMRI: resting state functional magnetic resonance imaging
rTMS: repetitive transcranial magnetic stimulation
SPIRIT: Standard Protocol Items: Recommendations for Interventional Trials
SUD: substance use disorder
VA: Department of Veterans Affairs
VistA: Veterans Health Information Systems and Technology Architecture
WHODAS 2.0: WHO Disability Assessment Schedule

## Data Availability

Data sharing is not applicable to this article as no data sets were generated or analyzed during this study. A deidentified, anonymized dataset will be created and shared. Raw and normalized data will be made available in the form of Microsoft Excel files. Magnetic resonance imaging images will be anonymized and made available through the Northwestern University Research Image Processing System. Final datasets will be made available as per the Edward Hines Jr VA Hospital local policy for long-term storage and access until enterprise-level resources become available. These data will be available on request by researchers and scientists in accordance with federal guidelines and the Edward Hines Jr VA Hospital local policy. The data provided will be sufficient for anyone to perform analogous or supplemental analyses that would permit the validation of the analysis and results. The sharing of data will enable others to evaluate the data and to validate and interpret the data independently. To enable replication and ensure transparency, statistical code complementary to datasets will be made available through the Federal Interagency Traumatic Brain Injury Research Informatics System.
