# Supplementary material for: A Customized Neural Transcranial Magnetic Stimulation Target for Functional Disability Among Veterans With Co-Occurring Alcohol Use Disorder and Mild Traumatic Brain Injury: Protocol for a Pilot Randomized Controlled Trial
Source: JMIR Res Protoc. 2025 Jun 23;14:e64909. doi: 10.2196/64909 (PMC12235201; doi:10.2196/64909)
Supplement: Multimedia Appendix 1 [file resprot_v14i1e64909_app1.pdf]

SUMMARY OF DISCUSSION:
A subcommittee of the Rehabilitation Research and Development Scientific Merit Review Board met in Plenary Session and reviewed the above proposal considering all internal and external reviews. This document summarizes the major points of the discussion concerning the proposed project. In any further development of this project, the investigator should consider carefully all the issues reflected in this Summary of Discussion as well as the more detailed comments in the individual critiques.

GENERAL COMMENTS:
- The investigators have been responsive to the reviewers (particularly around feasibility). The authors have outlined specific time-point and goals, have developed a less restrictive matching strategy for recruitment, and have given more details about human subjects and recruitment.
- The authors have tried to address the concerns that this work is very preliminary by providing data from Dr. Philip. Unfortunately, Dr. Herrold's pilot data is still being collected.
- It was unclear whether the neural targets determined from the fMRI task are based on associations with the baseline characteristics or with results from the fMRI task.
- The investigators did address some of the issues of feasibility raised about the phase that involves testing rTMS. However, there are still issues that linger. Fewer groups would require a smaller sample size (this is where pilot data might inform the research). There are confounds that can influence treatment outcome which is one of the limitations of doing work in comorbid conditions. The authors do not address this concern adequately. Finally, the authors have focused on a primary outcome of functional disability-some other outcomes that are also important clinically would strengthen the proposal.

SUGGESTIONS:
It was also noted that while the investigators did address the concern that the exclusion criteria for the control group were too restrictive, they continue to have a WHODAS exclusion score which should be reconsidered.

CONDITIONS:
WHODAS should be a co-variate not an exclusion criterion.

COMMENTS ON THE BUDGET:
No budgetary concerns.

ADMINISTRATIVE NOTE:
The presence of a condition(s) is not an indication that the application has been selected for funding. If a notice of intent to award is issued, then the condition(s) will need to be addressed as part of Just-in-Time (JIT).

DESCRIPTION (provided by applicant):
Alcohol use disorder (AUD) and mild traumatic brain injury (mTBI) impact functional abilities. AUD occurs in up to 35% of Veterans with mTBI. Evidence suggests that co-occurrence of AUD and mTBI (AUD+mTBI) leads to an exacerbation of brain dysfunction, symptom manifestation, and ultimately, functional disability. Alcohol- related characteristics are operationally defined per AUD symptoms and outcomes including, but not limited to, alcohol consumption, alcohol craving, and AUD severity. Repetitive transcranial magnetic stimulation (rTMS) is a non-invasive neuromodulatory treatment that will soon be a treatment option at 30 VAs nationwide. Preliminary rTMS efficacy is demonstrated for AUD alone and mTBI alone using a variety of neural targets. rTMS is, thus, a promising treatment for AUD+mTBI. The objectives of this study are to 1) identify neural targets (i.e. site of stimulation) associated with both alcohol-related characteristics and self-reported functional disability, and 2) assess preliminary efficacy and sustainability of a high frequency rTMS protocol applied to these customized neural targets relative to the commonly used left dorsolateral prefrontal cortex (DLPFC) site. Addressing these objectives are essential steps towards our long-term research goal [to customize

and clinically implement a rTMS treatment] that can improve brain function resulting in optimal recovery for Veterans with AUD+mTBI. To address the first study objective, Veterans will be recruited and classified into one of two groups based on structured-interviews, self-report measures, and neuropsychological assessments: 1) AUD+mTBI, and 2) [Veteran controls] without a history or symptoms of mTBI or AUD. Alcohol-related characteristics will be assessed through objective measures of alcohol use, self-report measures, and structured interviews. Self-reported functional disability will be assessed using the World Health Organization Disability Assessment Schedule 2.0 (WHODAS). Neuroimaging metrics will be assessed through a multi- modal, functional and structural Magnetic Resonance Imaging (MRI) scan. Participants will complete a functional MRI (fMRI) protocol where brain activation will be measured in response to viewing images related to alcohol, compared to neutral images. Advanced neuroimaging procedures to determine the structural integrity of white matter fibers in the brain and spontaneous activity in brain networks, a process called resting state functional connectivity (rsFC), will also be conducted. To address the second study objective, AUD+mTBI Veterans will receive rTMS at one site randomly assigned from a set of 4 sites: 3 customized neural targets identified in this study, and the commonly used left DLPFC. AUD+mTBI Veterans will complete 10 Placebo, then 10 Active rTMS sessions in a within-subjects design. Follow-up WHODAS assessments will occur at 2- weeks, 1-month and 6-months post-ACTIVE rTMS. Aim 1 will identify unique neural targets for rTMS to treat AUD+mTBI by determining which multi-modal neuroimaging metrics are most strongly associated with both alcohol-related characteristics and functional disability. Aim 2 will [test preliminary efficacy of high-frequency rTMS administered over the customized neural targets] to treat functional disability among Veterans with AUD+mTBI. Aim 3 will assess sustainability of rTMS effects on functional disability for Veterans with AUD+mTBI. We hypothesize that for Veterans with AUD+mTBI, there are neural substrates of AUD related to functional disability, and that neuromodulation of these substrates will be related to gains in functional disability. Our innovative approach represents an advancement in the field of neurorehabilitation because a neural target will be systematically defined, using multi-modal neuroimaging, prior to preliminary rTMS efficacy and sustainability testing. These steps are necessary to customize rTMS treatment for a population of Veterans with co-occurring conditions and unique health care needs. Thus, the outcomes of this research will optimize function for Veterans with AUD+mTBI.

PUBLIC HEALTH RELEVANCE:
The objectives of this VA Merit application are to identify a neural target unique to Veterans with co-occurring alcohol use disorder and mild traumatic brain injury (AUD+mTBI) and to test the efficacy of this target as a stimulation site for repetitive transcranial magnetic stimulation (rTMS) treatment to maximize functional recovery. rTMS will soon be a treatment option at 30 VAs nationwide and preliminary studies show promise for AUD and mTBI treatment. A better understanding of how AUD+mTBI impacts the brain needs to occur in order to advance rTMS to optimize function. This research is aligned with the VA RR&D’s mission to generate knowledge and innovations to advance the rehabilitative health and care of Veterans, to effectively integrate clinical and applied rehabilitation research, and translate research results into practice. This research is also aligned with the goal of the Psychological Health & Social Reintegration portfolio to develop interventions improving psychological health status of Veterans enabling them to function more fully in society.

CRITIQUE 1

Significance and Innovation: The proposed study is aligned with VA mission to promote treatments for health conditions that affect Veterans-with its emphasis on AUD and TBI. The plan to develop a customized protocol for rTMS for those with co-morbid AUD and mTBI is important clinically as this is a vulnerable population for which there are not adequate treatments and this population has high rate of other comorbid conditions (e.g. PTSD). The phased approach to determine neural targets is in line with a “personalized medicine” approach and has potential to influence the way treatment is delivered-this is particularly important given the VA emphasis on roll out of rTMS nationally.

The potential is somewhat mitigated by the fact that this is a very preliminary study. The evidence for rTMS particularly in addictive disorders and in comorbid conditions is not robust (decreasing craving for alcohol does not always translate to reducing consumption which is the focus of treatment). This was noted by previous reviewers-who requested more pilot data, but unfortunately more pilot data was unavailable (still being collected etc.). It is unclear whether neural targets as identified by fMRI may or may not translate to a therapeutic target for rTMS. It should be noted that even if the study is positive, this may not translate to an effective treatment since the primary outcome is functional disability and outcomes that are the target of treatment interventions (e.g. alcohol consumption is the most important AUD outcome) are not included.

Importance and Impact: If positive, this work has the potential to influence the treatment of Veterans who have this common comorbid condition. Even if negative, results from this study may facilitate the identification and understanding of potential neural targets and may lead to insight of the neurobiology of these disorders –this is still of interest to the field.

Contribution to VHA: Developing targeted treatment using rTMS has the potential to influence the way treatment is delivered-this is particularly important given the VA emphasis on roll out of rTMS nationally.

Methods (Data Analysis)**:** The first phase is collecting neuroimaging data –using indices of functional activation to alcohol cues, gray matter density and structural and resting state functional connectivity-analyzing this using of multilevel modeling. In addition, some assessments will be used as covariates-although it was not clear how these would be used. Some of these seem very reasonable covariates (e.g. TLFB- apparently using alcohol consumption data-although the authors do not outline how they plan to use it-quantity? Frequency?)-but with others it is harder to understand how the data will be used (e.g. ASI). It is also unclear whether the neural correlates will be determined by the results of the alcohol task or based on associating with functional disability based on the WHODAS. The second phase is testing preliminary efficacy of a high frequency rTMS (using customized targets or a standard approved target) with a primary outcome of functional disability (WHODAS). Having other outcomes even if they are just exploratory would be interesting-seems like this may be a “missed opportunity”.

Adequacy of Data: The investigators have designed a study that has adequate data to answer the primary Specific Aims. The baseline information and assessments are well thought out and will provide adequate data to understand the characteristics of the population that is participating in this study. The fMRI task and data collection are reasonable, and the investigators have experience in collecting and analyzing this data. The primary outcome for the rTMS feasibility study is functional disability. As mentioned above, it might have been nice to have some exploratory aims to collect other data on whether rTMS improves outcomes such as mood, alcohol consumption, etc.

Project Organization and Management: Dr. Herrold is the Principal Investigator (PI) responsible for the overall conduct of the project; she has adequate experience to conduct this work. She has included investigators with expertise in rTMS, fMRI, clinical trials and data collection/analysis. She also has included neuropsychologist and an addiction psychiatrist who have the clinical expertise for this trial.

Investigator Qualifications: The PI Dr. Amy Herrold, has assembled a strong team of investigators. They each bring expertise relevant and complementary expertise to this proposal. They include experts in neuro-rehabilitation (Herrold); health services and data analysis (Jordan); fMRI (Durazzo); Mixed effects models of neuroimaging data (Bhaumik); Parrish (Neuroimaging); as well as others on the team: neuropsychologist (Riordan) Addiction Psychiatrist (Ramic); expert in psychometric properties (Mallinson).

Facilities and Resources: There are adequate facilities to conduct this research-in including the Edward Hines VA, which is the primary site of the study has among other programs an HSR&D Service Centers of Excellence, a VA Polytrauma System of Care and the adequate rTMS equipment. There are also adequate facilities for fMRI. The study will also recruit from 3 Chicago area VA's greatly enhancing the ability of recruitment. It should be noted that how this will exactly be conducted was not clear from the proposal (e.g. is there a way to transport patients between facilities?).

Human Subjects: The inclusion/exclusion criteria are well thought out, and the risks/ benefits are appropriate. The investigators have ensured that adequate care is taken for protection of human subjects.

Critique of Vertebrate Animals Section: No comment.

	Yes	No
Research with vertebrate animals?		X

Biohazards and Radioisotopes: Not applicable.

Inclusion of Women, Minorities and Children: Recruiting Veterans from age 18-65, women and men included. Investigators will make an effort to recruit women from a Women's Health Clinic. The population will reflect the population of the patients treated at the 3 Chicago-based VA facilities.

Budget (unscored)**:** Budget is reasonable, and no issues identified.

Data Management and Access Plan (for data sharing, unscored)**:** Data management and access plan is acceptable for this proposal. No issues identified.

Overall Strengths: This proposal is an interesting and very clinically important phased study. The overall goal is to develop a targeted rTMS for Veterans with AUD and mTBI. The revised application is responsive to previous reviewer comments, particularly in the area of feasibility, subject recruitment and confounds among subjects (between the cases and the controls). The evaluation phased approach-to determine neural targets that might help identify specific treatment targets and finally the testing of these targets is an innovative approach. The research team is an excellent team of outstanding researchers with the expertise needed to conduct this research.

Overall Weaknesses: The protocol continues to be plagued by the fact that this is very preliminary work, and while the investigators have responded to this criticism, the lack of pilot data continues to be an issue. The authors similarly have responded to issues of recruitment and feasibility-by outlining a plan to recruit from other VA sites, but there are questions on how easy this will be-whether patients will be able to come from VA's that are geographically far from testing site and whether this will influence the subject pool. There are a number of confounding conditions that may influence results (e.g., comorbid mood disorders, PTSD). The investigators use a single blind design to compare subjects to placebo, but it is not clear if this is a "run in" design (where subjects have to meet criteria for entry again). There also continue to be 4 groups, which requires a large sample size-not sure if investigators considered limiting the specific treatment targets to the most likely (this is an area where preliminary data would have been helpful). Finally, it seems that there may be some "missed opportunities" here, in the collection of data usually collected in clinical trials (e.g. alcohol consumption, mood, symptoms of PTSD, etc.).

CRITIQUE 2

Significance and Innovation: This study proposes to identify optimal neural stimulation sites for high-frequency rTMS among those with combined mTBI and AUD using a combination of functional (task-based, resting state connectivity) and structural (gray matter volume, DTI) imaging measures that relate to alcohol-related characteristics (ARCs) and functional disability. It also proposes to preliminarily test the efficacy of these sites for treating functional disability against the typical left DLPFC site using a within-subjects design. Finally, it will test the stability of effects at 2-weeks, 1 month, and 6-months post-intervention.

This study would be the first to select customized neural targets for mTBI+AUD, the first rTMS study for AUD to include Veterans, and the second rTMS study for mTBI to include Veterans. This study ranks highly in terms of innovation, and the investigative team is commended for their efforts at increasing personalized medicine using careful measures of neural functioning.

Importance and Impact: Co-occurring mTBI and AUD are major public health problems. Among Veterans, 35% of those with mTBI have co-occurring TBI, and comorbidity is related to increased brain impairment and symptoms, and has a negative impact on rehabilitation efforts, relative to either condition alone (as demonstrated by the PI and others work). If successful, this study could improve personalized medicine and provide a supplemental or stand-alone treatment for those with mTBI-AUD.

Contribution to VHA: The VHA has initiated clinical rTMS use, having purchased 30 devices nationwide. Thus, if successful, the neural targets identified as part of this study could be disseminated as part of clinical protocols using rTMS.

Methods: Good justification is provided for inclusion of a healthy control group.

The group classification method is comprehensive and well-validated.

The plan to enroll 191 Veterans is more feasible than the original target of 270.

Adequacy of Data: The application provides good justification for use of the WHODAS as the primary outcome measure of functional disability. Other proposed self-report and behavioral measures are reliable and well-validated.

The preliminary data provides support for the feasibility of Aim 1. It shows differences between healthy controls and those with AUD+mTBI in regions that were related to ARCS and functional disability using task-based fMRI, resting state connectivity using a region-of-interest (ROI) approach, and gray matter density.

Preliminary data is now provided that demonstrates feasibility of performing rTMS in the proposed sample, collected by colleague Philip (who has included a letter of support). There is also now preliminary DTI data.

A strength of the recruitment method is use of a data repository maintained by the PI and co-I Pape, created for study recruitment purposes.

Project Organization and Management: The project organization and management plan is adequate.

Investigator Qualifications: The PI is a is a neuroscientist with a CDA-2 (ends 2019) award on a complimentary topic to the proposed study (that includes neuroimaging), with a strong track record of publications on the topic of mTBI and AUD. The study team exceptionally strong, with a history of collaboration.

Facilities and Resources: The facilities and resources appear adequate.

Human Subjects: The Human Subjects plan is adequate.

Critique of Vertebrate Animals Section: Not applicable.

	Yes	No
Research with vertebrate animals?		X

Biohazards and Radioisotopes: Not applicable.

Inclusion of Women, Minorities and Children: Children are not included as part of this project. Women will be targeted via the Women's Health Clinic at Hines and work with the VA Women's Health Practice Based Research Network

Budget (unscored)**:** No issues.

Data Management and Access Plan: The data sharing plan is adequate.

Overall Strengths: This is a nicely revised application that addresses an important public health issue, with the potential to impact the care of Veterans with comorbid mTBI and AUD. The applicants have done an excellent job addressing the reviewers' critiques, including reducing the number of individuals to be recruited (enhancing feasibility), increasing homogeneity of the sample by restricting recruitment to Veterans and clarifying inclusion/exclusion criteria, and clarifying their plan for a future trial using the best stimulation site identified.

The methodology is rigorous and innovative.

The study team is strong with a history of collaboration.

Overall Weaknesses: No major weaknesses.

CRITIQUE 3

Significance and Innovation: This critique is based on the methodology only including the concerns noted on the prior review.

Importance and Impact: No concerns.

Contribution to VHA: No concerns.

Methods (Data Analysis)**:** The prior concerns related to recruitment and inclusion / exclusion criteria by this reviewer have been adequately addressed. One concern remaining is the new requirement of a WHODAS score >= 10 for the Veteran control group and lack of rationale for this added exclusion criterion which, like the previous exclusions, may lead to lack of representativeness of the control group.

Adequacy of Data: No concerns.

Project Organization and Management: No concerns.

Investigator Qualifications: No concerns.

Facilities and Resources: No concerns.

Human Subjects: No concerns.

Critique of Vertebrate Animals Section: No concerns.

	Yes	No
Research with vertebrate animals?		X

Biohazards and Radioisotopes: No concerns.

Inclusion of Women, Minorities and Children: No concerns.

Budget (unscored)**:** No concerns.

Data Management and Access Plan (for data sharing, unscored)**:** No comment.

Overall Strengths: No concerns.

Overall Weaknesses: No concerns.

MEETING ROSTER

Behavioral Health & Social Reintegration
Rehabilitation Research and Development Parent IRG
Office of Research & Development
RRD4
02/28/2019

CHAIRPERSON(S)

HUNT, PETER C., PHD
DIRECTOR
BUSINESS MODEL DEVELOPMENT
ALLOCATION RESOURCE CENTER
BRAINTREE, MA 02184

MEMBERS

ACION, LAURA, PHD *
ASSOCIATE RESEARCHER
IOWA CONSORTIUM FOR SUBSTANCE ABUSE RESEARCH
AND RESEARCH AND EVALUATION
UNIVERSITY OF IOWA
IOWA CITY, IA 52242

ANDERSON, BRIAN J., MD *
ASSOCIATE MEDICAL DIRECTOR
MEDICAL INTENSIVE CARE UNIT
PULMONARY, ALLERGY, AND CRITICAL CARE DIVISION
HOSPITAL OF THE UNIVERSITY OF PENNSYLVANIA
PHILADELPHIA, PA 19104

ASTON, ELIZABETH, PHD *
ASSISTANT PROFESSOR
BEHAVIORAL AND SOCIAL SCIENCES
BROWN UNIVERSITY
PROVIDENCE, RI 02903

BEALS, KIM PHD *
ASSISTANT PROFESSOR
DEPARTMENT OF SPORTS MEDICINE AND NUTRITION
SCHOOL OF HEALTH & REHABILITATION SCIENCES
UNIVERSITY OF PITTSBURGH
PITTSBURGH, PA 15203

BENNETT, MELANIE E., PHD *
DIRECTOR
VISN 5 MIRECC ADVANCED FELLOWSHIP
BALTIMORE VA MEDICAL CENTER
DIRECTOR, EDUCATION CORE
UNIVERSITY OF MARYLAND SCHOOL OF MEDICINE
BALTIMORE, MD 21201

BUSH, HEATHER M., PHD *
PROFESSOR AND CHAIR
DEPARTMENT OF BIOSTATISTICS
COLLEGE OF PUBLIC HEALTH
UNIVERSITY OF KENTUCKY
LEXINGTON, KY 40536

CATER, JANET PHD, PHD *
VOCATIONAL REHABILITATION COUNSELOR
MUSKOGEE VA REGIONAL OFFICE
MUSKOGEE, OK 74401

COLLINS, JAMIE E., PHD *
BIOSTATISTICIAN
ORTHOPAEDIC AND ARTHRITIS CTR FOR OUTCOMES RES
BRIGHAM AND WOMEN'S HOSPITAL
BOSTON, MA 02115

COPELAND, LAUREL A, PHD
RESEARCH SCIENTIST
VA CENTRAL WESTERN MASSACHUSETTS HEALTHCARE
LEEDS, MA 01053

HOPKINS, RAMONA O, PHD *
DIRECTOR
LDS HOSPITAL
CRITICAL CARE MEDICINE
SALT LAKE CITY, UT 84143

KING, HEATHER A, PHD *
RESEARCH HEALTH SCIENTIST
DURHAM VA MEDICAL CENTER
HEALTH SERVICES RESEARCH AND DEVELOPMENT
ASSISTANT PROFESSOR
DUKE UNIVERSITY, DEPT OF MEDICINE
DURHAM, NC 27705

KUHN, ERIC ROLAND, PHD *
CLINICAL ASSOCIATE PROFESSOR (AFFILIATED)
NATIONAL CTR FOR PTSD DISSEMINATION AND TRAINING
CLINICAL ASSISTANT PROFESSOR
DEPT OF PSYCHIATRY AND BEHAVIORAL SCIENCES
STANFORD UNIVERSITY SCHOOL OF MEDICINE
MENLO PARK, CA 94025

METE, MIHRIYE, PHD
BIOSTATISTICS MANAGER
DEPT OF BIOSTATISTICS AND BIOINFORMATICS
MEDSTAR HEALTH RESEARCH INSTITUTE
CENTER FOR TRAUMA AND THE COMMUNITY
GEORGETOWN UNIVERSITY MEDICAL CENTER
HYATTSVILLE, MD 20782

ORR, JOSEPH M., PHD *
ASSISTANT PROFESSOR
DEPARTMENT OF PSYCHOLOGICAL AND BRAIN SCIENCES
TEXAS A&M INSTITUTE FOR NEUROSCIENCE
TEXAS A&M UNIVERSITY
COLLEGE STATION, TX 77843

PETRAKIS, ISMENE L., MD *
CHIEF OF PSYCHIATRY
DEPARTMENT OF PSYCHIATRY
SCHOOL OF MEDICINE
YALE UNIVERSITY
WEST HAVEN, CT 06516

PRUIKSMA, KRISTI, PHD *
ASSISTANT PROFESSOR
UNIVERSITY OF TEXAS HEALTH SCIENCE CENTER
SAN ANTONIO, TX 78229

RESNIK, LINDA J., PHD *
RESEARCH CAREER SCIENTIST
PROFESSOR
DEPT OF HEALTH SERVICES POLICY AND PRACTICE
BROWN UNIVERSITY
PROVIDENCE, RI 02906

SCHER, ANN I, PHD *
PROFESSOR
DEPT OF PREVENTIVE MEDICINE AND BIOMETRICS
UNIFORMED SERVICES UNIV OF HEALTH SCI
BETHESDA, MD 20814

TEUFEL, JAMES AUGUST, PHD *
DIRECTOR AND ASSISTANT PROFESSOR
PUBLIC HEALTH SALLY MIKSIEWICZ CENTER
BETHLEHEM, PA 18018

TOROUS, JOHN, MD *
DIRECTOR
DIGITALPSYCH.ORG
BETH ISRAEL DEACONESS MEDICAL CENTER
BOSTON, MA 02115

WEISENBACH, SARA L., PHD *
ASSISTANT PROFESSOR
DEPARTMENT OF PSYCHIATRY
UNIVERSITY OF UTAH
SALT LAKE CITY, UT 84112

WINTER, LARAINE, PHD *
ASSISTANT DIRECTOR
THOMAS JEFFERSON UNIVERSITY
RESEARCH PSYCHOLOGIST
PHILADELPHIA RESEARCH AND EDUC FOUNDATION
PHILADELPHIA, PA 19107

WISNIEWSKI, STEPHEN R, PHD *
PROFESSOR
DEPARTMENT OF EPIDEMIOLOGY
EPIDEMIOLOGY DATA CENTER
UNIVERSITY OF PITTSBURGH
PITTSBURGH, PA 15260

ZIGLER, CHRISTINA PHD *
BIOSTATISTICIAN
HUMAN ENGINEERING RESEARCH LABORATORIES
VA PITTSBURGH HEALTHCARE SYSTEM
DEPT OF PHYSICAL MEDICINE & REHABILITATION
UNIVERSITY OF PITTSBURGH
PITTSBURGH, PA 15260

SCIENTIFIC REVIEW OFFICER

ROBINSON, CENDRINE, PHD
SCIENTIFIC PROGRAM MANAGER
DEPARTMENT OF VETERAN AFFAIRS
OFFICE OF RESEARCH AND DEVELOPMENT
REHABILITATION RESEARCH AND DEVELOPMENT SERVICE
WASHINGTON, DC 20420

* Temporary Member. For grant applications, temporary members may participate in the entire meeting or may review only selected applications as needed.

Consultants are required to absent themselves from the room during the review of any application if their presence would constitute or appear to constitute a conflict of interest.
